# A path model examination: maternal anxiety and parenting mediate the association between maternal adverse childhood experiences and children's internalizing behaviors

**DOI:** 10.1017/S0033291721001203

**Published:** 2023-01

**Authors:** Emily W. Shih, Shaikh I. Ahmad, Nicole R. Bush, Danielle Roubinov, Fran Tylavsky, Carolyn Graff, Catherine J. Karr, Sheela Sathyanarayana, Kaja Z. LeWinn

**Affiliations:** 1 University of California, San Francisco, San Francisco, CA, USA; 2 University of Tennessee, Knoxville, TN, USA; 3 University of Washington, Seattle, WA, USA; 4 Seattle Children's Research Institute, Seattle, WA, USA

**Keywords:** ACEs, adverse childhood experiences, child psychopathology, internalizing behaviors, maternal anxiety, parental psychopathology, parenting

## Abstract

**Background:**

Children of mothers with adverse childhood experiences (ACEs) are at increased risk for developmental problems. However, the mechanisms through which a mother's experience of ACEs are transmitted to her offspring are understudied. The current study investigates potential modifiable mediators (maternal psychopathology and parenting) of the association between maternal ACEs and children's behavioral problems.

**Methods:**

We utilized data from a pregnancy cohort study (*N* = 1030; CANDLE study) to investigate longitudinal associations between maternal ACEs, postpartum anxiety, observed parenting behavior, and child internalizing behaviors (*mean*_age_ = 4.31 years, *s.d.*_age_ = 0.38) in a racially diverse (67% Black; 33% White/Other) sample. We used structural equation modeling to test for direct associations between maternal ACEs and children's internalizing behaviors, as well as indirect associations via two simple mediations (maternal anxiety and parenting), and one serial mediation (sequence of maternal anxiety to parenting).

**Results:**

Simple mediation results indicated that maternal anxiety and cognitive growth fostering behaviors independently mediated the association between maternal ACEs and child internalizing. We observed no evidence of a serial mediation from ACEs to internalizing via the effects of maternal anxiety on parenting.

**Conclusions:**

This study supports and refines extant literature by confirming the intergenerational association between maternal ACEs and child internalizing behaviors in a large, diverse sample, and identifies potential modifiable mediators: maternal anxiety and parenting behaviors related to fostering cognitive development. Findings may inform interventions targeting mothers who have experienced ACEs and suggest that providing support around specific parenting behaviors and addressing maternal anxiety may reduce internalizing behaviors in children.

Depression and anxiety are the most common mental health disorders among children and adolescents and rates appear to be increasing (Ghandour et al., 2019). Evidence suggests that internalizing behaviors in childhood predict development of future depression and anxiety (Gotlib & Hammen, 2008). Thus, it is important to identify the modifiable factors in early childhood that may be targeted in intervention efforts to reduce childhood behavioral problems. Maternal experiences of childhood adversity have long-term health impacts, and a growing body of research supports intergenerational associations with their children's health and risk for later psychopathology (Letourneau et al., 2019; Lê-Scherban, Wang, Boyle-Steed, & Pachter, 2018). Adverse childhood experiences (ACEs) are defined as a set of exposures to abuse, neglect, and household dysfunction prior to 18 years of age. Despite the robust literature surrounding the etiology and developmental mechanisms of emotional and behavior problems in children, only a few studies have examined the intergenerational associations between maternal ACEs and child psychopathology in well-characterized, longitudinal studies. Research suggests that children of mothers who have been exposed to ACEs are at increased risk of poor health and developmental outcomes (Folger et al., 2018; Gavin, Nurius, & Logan-Greene, 2012), including social, emotional, and behavioral problems, as well as increased likelihood of parent–child relationship difficulties (Berthelot et al., 2015; McDonnell & Valentino, 2016). However, research examining the specific pathways by which maternal ACEs confer greater risk for child psychopathology is lacking. Understanding these potential mechanisms would inform more tailored intervention and prevention efforts designed to break the intergenerational cycle of risk for poor mental health. Two key modifiable pathways that have been proposed regarding the intergenerational association between maternal ACEs and child psychopathology are maternal psychopathology (Cooke, Racine, Plamondon, Tough, & Madigan, 2019; Letourneau et al., 2019; Schickedanz, Halfon, Sastry, & Chung, 2018) and parenting practices (Stepleton et al., 2018). Although ACEs are linked to maternal depression, associations with maternal anxiety are less well studied, despite the fact that anxiety disorders are the most common mental health problem among adults with prevalence rates twice as high in women compared to men (Bandelow & Michaelis, 2015). We address this gap in the literature by investigating whether maternal ACEs are associated with children's internalizing behaviors via maternal anxiety and parenting in a racially diverse longitudinal study of over 1000 children from the southern region of the USA.

## Potential mediators of maternal ACEs and child internalizing behaviors

### Maternal anxiety

A plausible mechanism linking maternal ACEs and child behavior is through maternal anxiety. Early childhood adversity is strongly associated with adult anxiety (Elmore & Crouch, 2020), and maternal anxiety is potentially detrimental for children because it can influence a number of factors that shape a child's environment, such as parent–child interactions (PCIs) and familial conflict (Muzik et al., 2017; Oyserman, Mowbray, Meares, & Firminger, 2000). For example, maternal anxiety is associated with reduced productive parental engagement (Woodruff-Borden, Morrow, Bourland, & Cambron, 2002), warmth, granting of autonomy, and increased catastrophizing (Whaley, Pinto, & Sigman, 1999); however, results are inconsistent with some studies finding no association (Drake & Ginsburg, 2011; Ginsburg, Grover, & Ialongo, 2005). Differences in methodology, informants, and composition of the sample used across studies might explain the contradictory results (Ginsburg et al., 2005), and additional research using high quality, observed parenting data is needed for clarification. Evidence suggests that anxious mothers may be more withdrawn (Woodruff-Borden et al., 2002) and thus less sensitive to their child's level of development and less able to adjust their behaviors accordingly (scaffolding; Vygotsky, 1980); therefore, we expect that anxious mothers would have more difficulty engaging in positive PCIs, which would subsequently effect children's internalizing behaviors. However, to our knowledge, few studies have examined associations between maternal ACEs and positive PCIs, and no studies to date have examined whether maternal anxiety may mediate this association.

### Parenting

In addition to the increased risk of anxiety and other psychopathology, exposure to adversity early in life may also directly affect parenting behaviors that are central to behavioral development (Lomanowska, Boivin, Hertzman, & Fleming, 2017; Savage, Tarabulsy, Pearson, Collin-Vézina, & Gagné, 2019). Mothers who experience childhood maltreatment may develop fewer resources to cope with parenting challenges and subsequently adopt more problematic parenting behaviors. For example, maternal ACEs have been associated with impairments in recognition of emotions and emotion regulation, which in turn are associated with problematic parenting behaviors (Kolomeyer, Renk, Cunningham, Lowell, & Khan, 2016). Additionally, parents who suffered from ACEs report feeling less confident as parents, have difficulty setting boundaries, act more permissively, prematurely promote children's autonomy, and rely more on their children for emotional support (Banyard, 1997; DiLillo & Damashek, 2003). Questions remain, however, regarding the importance of positive PCIs in this population; given that mothers who have experienced ACEs are more likely to have poorer quality dialogs with their children and show less sensitive guidance (Visser et al., 2016), these interactions are worthy of further study. For example, how parents scaffold their children's social-emotional growth (e.g. emotion regulation) and cognitive growth (e.g. problem-solving) are important contributors to children's adjustment (Johnson, Hawes, Eisenberg, Kohlhoff, & Dudeney, 2017). Although these parenting practices are also likely to be affected by maternal ACEs, few studies have examined this relation directly. Furthermore, to our knowledge, no studies have examined whether there is a meaningful pathway from maternal ACEs, through positive PCIs, to child internalizing behaviors.

Collectively, prior research suggests that a comprehensive, longitudinal evaluation of how these key factors (maternal ACEs, maternal anxiety, and positive PCIs) that contribute to a child's developmental environment work together and, in turn, influence child internalizing symptoms, is needed. To our knowledge, only one well-powered study has assessed the intergenerational transmission of maternal ACEs on child psychopathology via pathways of maternal psychopathology; however, this study was conducted in a white, upper-middle class Canadian sample that is unlikely generalizable to US populations (Letourneau et al., 2019). The current study attempts to address this gap in the literature.

## Current study

We utilized the Conditions Affecting Neurocognitive Development and Learning in Early Childhood (CANDLE) study, a pregnancy cohort of 1503 mother–child dyads, to investigate longitudinal associations between maternal ACEs, maternal anxiety, parenting during toddlerhood, and internalizing behaviors at child ages 4–6. We chose to focus our investigation on the toddlerhood and early childhood developmental periods, given that children are especially dependent on their parents for emotional support and families may be more malleable and responsive to intervention efforts in this developmental stage (Brooks-Gunn, 2003; Kingston & Tough, 2014). The CANDLE study is uniquely suited to address limitations in prior studies given its longitudinal design and a large, racially diverse (65% Black; 35% White/Other) sample from the urban south, where many families experience high levels of multiple forms of adversity. Furthermore, the CANDLE study is distinctive in that it includes an observed measure of parenting behavior using the NCAST Parent–Child Interaction (PCI) Teaching Scale assessed by trained behavioral coders, allowing us to rigorously estimate associations with parenting in a large diverse sample.

In the current study, we tested the direct associations between maternal ACEs and children's internalizing behaviors, as well as whether two potential mechanisms, maternal anxiety and parenting, independently mediate this association. We also tested whether maternal ACEs predict children's internalizing behaviors via increased maternal anxiety and its subsequent influences on parenting practices (i.e. serial mediation of the maternal ACEs and child internalizing behaviors association). Using the PCI, we examined associations with three indicators of positive parenting: a global indicator of positive PCIs using the total caregiver scale as well as two subscales that capture maternal scaffolding of socioemotional and cognitive growth. We tested three hypotheses. First, we hypothesized a positive association between maternal ACEs and child internalizing behaviors. Second, both maternal anxiety and maternal parenting will independently mediate the relation between maternal ACEs and child internalizing behaviors. Specifically, we hypothesized that maternal ACEs would be positively associated with maternal anxiety and subsequently, more child internalizing behaviors; we also anticipated that maternal ACEs would be associated with reduced positive parenting and subsequently, more child internalizing behaviors. Third, we hypothesized that maternal anxiety and positive parenting would serially mediate positive associations between maternal ACEs and child internalizing behaviors, with maternal ACEs positively associated with maternal anxiety, which would lead to fewer positive parenting practices, and ultimately, increased child internalizing behaviors. We hypothesized the same direction of association for each of the three indicators of parenting practices (positive PCI, socioemotional scaffolding, and cognitive scaffolding).

## Method

### Participants

The CANDLE study was designed to determine the pre- and postnatal factors that impact neurocognitive development (http://www.candlestudy.com) with a robust, longitudinal design. Between 2006 and 2011, CANDLE enrolled 1503 healthy women during their second trimester of pregnancy. Women were eligible for the study if they were between 16 and 40 years old, residents of Shelby County, TN, proficient in English, and 16–28 weeks of gestation with a low medical risk singleton pregnancy. The University of Tennessee Health Science Center Institutional Review Board approved the study.

### Procedure and measures

The current study uses data collected from mother–child dyads from pregnancy through the child 4–6-year clinic visit. Data were gathered at multiple time points and settings (online Supplementary Table S1): two pre-natal clinic visits (enrollment, third trimester), one hospital visit (birth), and four clinic visits (1-year, 2-year, 3-year, and 4–6 year). Demographic surveys and a battery of tests and questionnaires were administered at each time point (LeWinn, Bush, Batra, Tylavsky, & Rehkopf, 2020; Sontag-Padilla, Burns, Shih, & Griffin, 2015).

#### Primary exposure

*Maternal ACEs.* The Traumatic Life Events Questionnaire (TLEQ) was collected at the third trimester prenatal clinic visit (Kubany et al., 2000). This self-report measure consists of 21 items, three of which are in reference to traumatic experiences that occurred during childhood. The three items assess experiences of sexual abuse before age 13, experiences of physical abuse while growing up, and experiences of witnessing family violence while growing up. A count of the number of childhood adversities experienced was created (0–3; 0 as the lowest and 3 as the highest possible score), as in prior published studies using the CANDLE study (Adgent et al., 2019; Pilkay, Combs-Orme, Tylavsky, Bush, & Smith, 2020; Slopen et al., 2018; Steine et al., 2020).

#### Mediators

*Maternal anxiety.* The 53-item Brief Symptom Inventory is a reliable and valid measure of psychological symptoms (Derogatis & Melisaratos, 1983). Responses are on a 5-point Likert-type scale, ranging from 0 (not at all) to 4 (extremely). The *T*-score for the Anxiety subscale, which consisted of 6-items and was collected during the intervening post-natal clinic visit time points (age 1, 2, 3; *α* > 0.77), was used as the measure of maternal anxiety. To obtain a more comprehensive assessment of maternal anxiety that captured symptoms across the child's early life, we averaged the three time points creating a composite maternal anxiety measure.

*Parenting.* The NCAST Parent–Child Interaction (PCI) Teaching Scale is a 73-item measure of parent and child behaviors that are rated immediately following observed mother–child interactions (Oxford & Findlay, 2013). The task was administered by a trained examiner, who had participated in PCI Teaching Scale training semi-annually and maintained reliability of 85% or higher (as verified by the Parent–Child Relationship Programs). Mothers are observed engaging with their child and teaching them during a pre-selected activity that the child has not yet mastered (e.g. stringing together a line of beads), which is designed to provoke minor levels of stress. Such interactions allow observation of adaptive patterns between mother and child outside of their typical interactive routines. Our primary analyses focus on the PCI Teaching Scale total caregiver score, which combines information from all the PCI subscales (sensitivity to cues, response to child's distress, social-emotional growth fostering, and cognitive growth fostering); we consider this total score to be a global indicator of positive PCIs (at age 2, *α* = 0.85; at age 3, *α* = 0.84). In secondary analyses, we focused on the social-emotional growth fostering (at age 2, *α* = 0.63; at age 3, *α* = 0.61), and cognitive growth fostering (at age 2, *α* = 0.70; at age 3, *α* = 0.68) subscales. These two subscales provide a measure of a mother's ability to engage the child in activities that stimulate social-emotional and cognitive growth using age appropriate language and motivators (Oxford & Findlay, 2013), and best reflected our interests around maternal scaffolding behaviors. The cognitive growth fostering subscale includes behaviors where parents were facilitating learning opportunities by focusing the child's attention or providing task instructions. Example items include ‘Caregiver focuses attention and child's attention on the task during most of the teaching’ and ‘Caregiver uses both verbal description and modeling simultaneously in teaching any part of the task’. The social-emotional growth fostering subscale includes behaviors such as playing affectionately, engaging in social interactions, and providing appropriate social reinforcement of desirable behaviors. Example items include ‘Caregiver positions self face-to-face with the child during the teaching interaction’ and ‘Caregiver laughs or smiles at child during the teaching interaction’. Items are scored yes (1 point) when a behavior is observed and no (0 points) when a behavior is not observed. All parenting measures were administered at both the age 2 and 3 time points, and thus, were averaged to create a more robust measure of maternal parenting behaviors over time.

The reliability for the NCAST Parent–Child Interaction Scale is established through the NCAST Office at the University of Washington. A certified instructor (co-author Dr Carolyn Graff) trained the study examiners on the PCI Teaching Scale. At the end of training, each coder observed and scored five videotaped caregiver–child dyads. These videotaped interactions were prepared by the NCAST Programs. The intercoder reliability was determined by how much the learners’ ‘Yes’ and ‘No’ scores on the PCI Teaching Scale aligned with the scores established by the NCAST Office for the videotaped dyads. All the coders were required to maintain reliability of 85% or higher, and were re-assessed and (if necessary) re-trained every 6 months.

*Outcome: child internalizing behaviors.* Child internalizing behaviors were measured using maternal report on the Child Behavior Checklist (CBCL) (Achenbach & Rescorla, 2000) when children were 4–6 years old. The CBCL is a widely used 99-item questionnaire that assesses behavioral and emotional problems in the past 6 months (Rescorla et al., 2011). Items are scored on a 3-point Likert scale (0 = absent, 1 = occurs sometimes, 2 = occurs often) across the following syndrome scales: Emotionally Reactive, Anxious/Depressed, Somatic Complaints, Withdrawn, Attention Problems, Aggressive Behavior, and Sleep Problems. Given our specific research interests, we focused on the broadband, total internalizing score (*α* = 0.85), which is a sum of all the internalizing behaviors subscales (anxiety, anxiety/depression, withdrawn, and affective problems).

### Statistical analyses

All SEM analyses were conducted using R (RStudio version 1.2.5033). Study variables were first examined for normality and the presence of outliers. Hypothesized pathways were tested using the ‘Lavaan’ package and subsequent analyses used full information maximum likelihood (FIML) with a missing at random assumption. Multiple fit indices were used to provide a more conservative and reliable evaluation of the model, including the comparative fit index (CFI), the standardized root mean square residual (SRMR), and the root mean square error of approximation (RMSEA). The following cut-off criteria were used to assess the fit between hypothesized models and the data: CFI ⩾ 0.90, SRMR ⩽ 0.08; RMSEA ⩽ 0.06 (Hu & Bentler, 1999). Reported path coefficients are standardized.

We estimated three separate models examining: (1) maternal anxiety and positive PCIs as mediators, (2) maternal anxiety and cognitive growth fostering as mediators, and (3) maternal anxiety and social-emotional growth fostering as mediators. To examine the prerequisites for potential mediation, we first tested the direct association between maternal ACEs and child internalizing behaviors controlling for child age (at the 4–6 year visit), sex, and mother's education. We considered child age and sex as covariates in our analyses because they are associated with parenting behaviors (Chaplin, Cole, & Zahn-Waxler, 2005). We further included maternal education given strong links with parenting behaviors associated with language enrichment and growth (Lanza, Rhoades, Greenberg, & Cox, 2011). Paths controlling for child sex, age, and maternal education were included leading to the outcome variable. Given the extant literature supporting differential parenting for boys and girls (Fivush, 1989; Fivush, Brotman, Buckner, & Goodman, 2000), a path toward parenting measures controlling for child sex was also included. Although we acknowledge that externalizing and internalizing behaviors are correlated in this age group, we do not adjust for externalizing behaviors in our analyses, as our aim was not to examine associations between maternal ACEs and child internalizing behaviors above and beyond associations related to externalizing behaviors (Brock & Kochanska, 2015; Raver, Roy, Pressler, Ursache, & Charles McCoy, 2017; Wagner, Propper, Gueron-Sela, & Mills-Koonce, 2016).

## Results

### Preliminary analyses

**Fig. 1.**
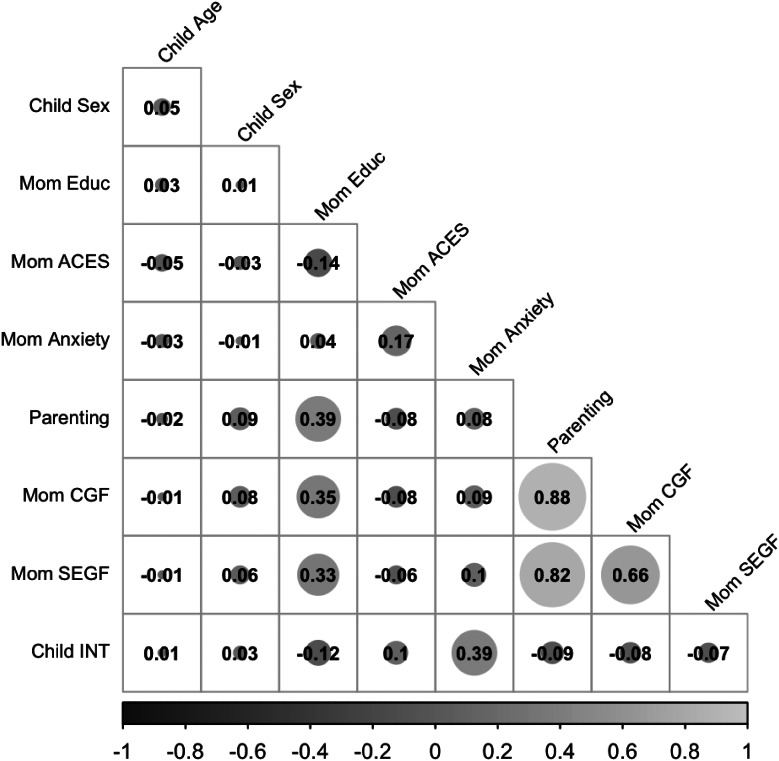
Bivariate Spearman correlations among primary study variables. ACEs, adverse childhood experiences; CGF, cognitive growth fostering; SEGF, social-emotional growth fostering; INT, internalizing behaviors.

Participants in the final analytical sample had complete data on our outcome measure, resulting in a sample of *N* = 1030 (3.76–5.98 years; mean *age* = 4.31 years, s.d. = 0.38; 513 boys). Data for the full and analytical sample are comparable and descriptive characteristics are presented in Table 1. Sixty-seven percent of the analytical sample identified as Black and 33% as White/Other. Forty-two percent of mothers reported having a college degree or higher. Twenty-four percent of mothers experienced one ACE, and 12% of mothers experienced two or more incidents (Table 2). Correlations between study variables were small to moderate (Fig. 1). Of note, maternal anxiety was positively correlated with child internalizing behavior (*r* = 0.39, *p* < 0.05).

**Table 1.** *t* test between the analytical sample and the full cohort among primary study predictors

	Full sample			Analytical sample		
Variables	*M*	s.d.	*n*	*M*	s.d.	*n*
Child age	4.43	0.63	1156	4.31	0.38	1030
Child race	1.51	1.01	1365	1.49	1.01	1021
Gender	1.50	0.50	1462	1.50	0.50	1030
Maternal education	3.27	4.18	1151	3.28	4.41	1025
Maternal ACEs	0.52	0.78	1361	0.52	0.79	971
Maternal anxiety	43.4	6.70	901	43.6	6.69	806
Maternal total caregiver subscale	40.0	5.03	936	40.0	5.04	821
Maternal cognitive growth fostering	12.7	2.17	936	12.8	2.15	821
Maternal social emotional growth fostering	8.66	1.50	936	8.65	1.49	821

**Table 2.** Demographic and descriptive characteristics

	*M*	s.d.	% (*N* = 1030)
Child age at outcome	4.3	0.4	–
Child gender			
Female	–	–	49.8
Male	–	–	50.2
Child race			
Black	–	–	66.5
White	–	–	28.2
Asian	–	–	0.7
Other	–	–	3.8
Unknown	–	–	0.9
Maternal education			
Less than high school education	–	–	5.2
GED/high school degree	–	–	39.2
Technical school	–	–	13.0
College degree	–	–	25.0
Professional degree	–	–	16.8
Unknown	–	–	0.7
Maternal ACEs			
No traumatic incident	–	–	59.2
Count of 1 incident	–	–	23.7
Count of 2 incidents	–	–	8.6
Count of 3 incidents	–	–	3.0
Did not respond	–	–	5.7
Maternal anxiety (BSI)	43.6	6.7	–
PCI Teaching Scale total caregiver subscale	40.0	5.0	–
PCI Teaching Scale cognitive growth fostering	12.8	2.2	–
PCI Teaching Scale social emotional growth fostering	8.7	1.5	–
CBCL child internalizing symptoms	6.2	6.1	–

ACEs, adverse childhood experiences; BSI, Brief Symptom Inventory; PCI, parent–child interaction; CBCL, Child Behavior Checklist.

### Primary analyses

**Fig. 2.**
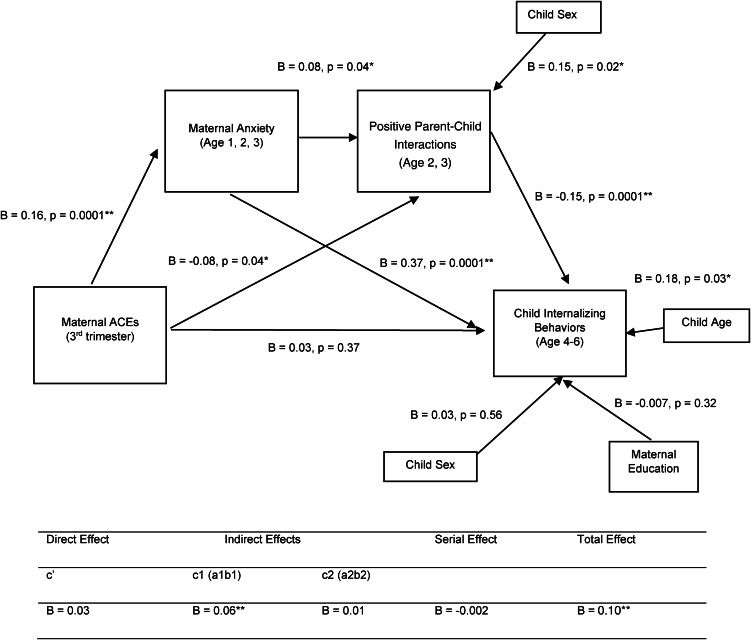
Mediation model. This model was conducted on the analytical sample (*N* = 1030), with variables imputed via FIML. Standardized path coefficients are reported. Controlling for child age, sex, and maternal education, results indicate a significant indirect effect from maternal ACEs to child internalizing problems through maternal anxiety. *p < 0.05; **p < 0.01; ***p < 0.001. ACEs, adverse childhood experiences.

We first estimated the model investigating maternal anxiety and positive parent–child interactions (PCI total caregiver subscale) as mediators of maternal ACEs and children's internalizing behaviors. Model fit was adequate according to recommendations for cut-off points (Hu & Bentler, 1999); SRMR = 0.03, RMSEA = 0.05 [0.03–0.08], CFI = 0.93. We observed a small but significant, direct association between maternal ACEs and child internalizing behaviors (*B* = 0.10, *p* = 0.002 [0.04–0.16]) (see Fig. 2), which was smaller in magnitude and no longer significant (*B* = 0.03, s.e. = 0.03, *p* = 0.37 [−0.03 to 0.09]) when maternal anxiety and positive PCIs were included in the model. The indirect effect of maternal ACEs on children's internalizing via a pathway of maternal anxiety was significant (*B* = 0.06, s.e. = 0.01, *p* < 0.001 [0.03–0.09]). However, the indirect effect via positive PCIs was not significant (*B* = 0.01, s.e. = 0.006, *p* = 0.065 [−0.001 to 0.02]). We observed no evidence of serial mediation (i.e. a pathway mediated by maternal anxiety to positive PCIs) (*B* = −0.002, s.e. = 0.001, *p* = 0.085 [−0.004 to 0.0001]) (i.e. Figure 2). The full model accounted for 16% of the variance in internalizing behaviors.

**Fig. 3.**
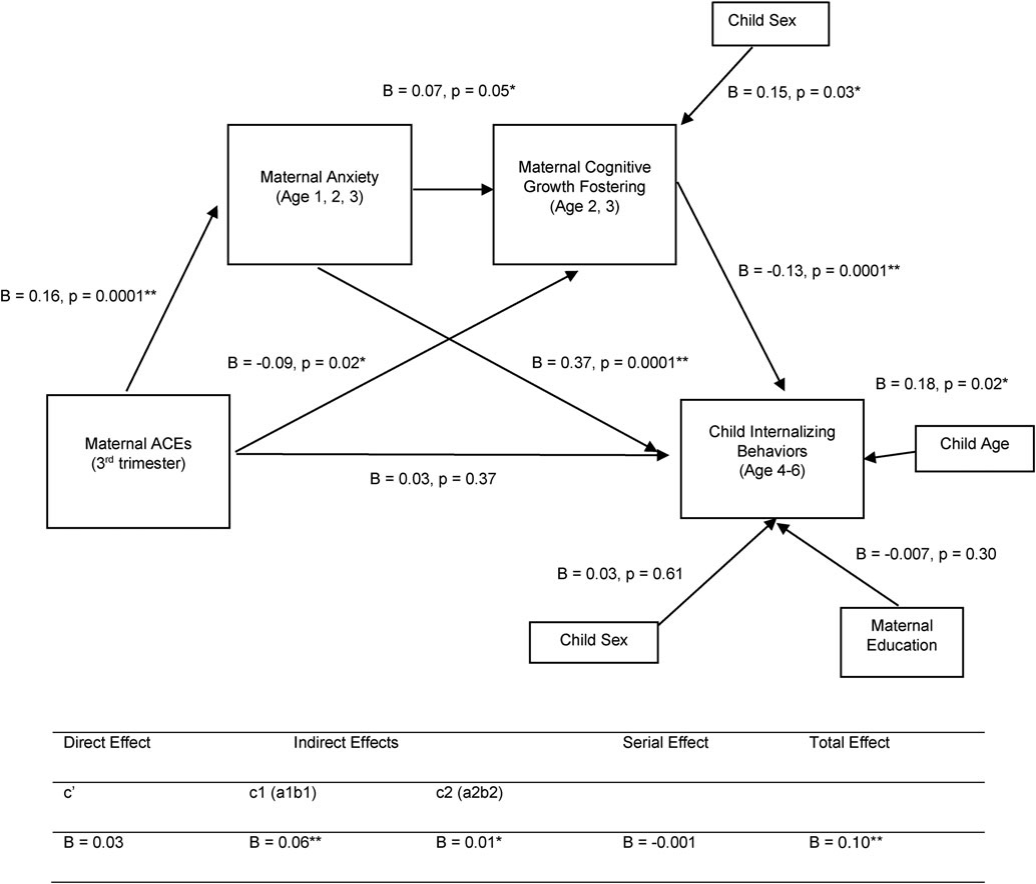
Mediation model. This model was conducted on the analytical sample (*N* = 1030), with variables imputed via FIML. Standardized path coefficients are reported. Controlling for child age, sex, and maternal education, results indicate (1) a significant indirect effect from maternal ACEs to child internalizing problems through maternal anxiety and (2) a significant indirect effect from maternal ACEs to child internalizing problems through maternal cognitive growth fostering. *p < 0.05; **p < 0.01; ***p < 0.001. ACEs, adverse childhood experiences.

**Fig. 4.**
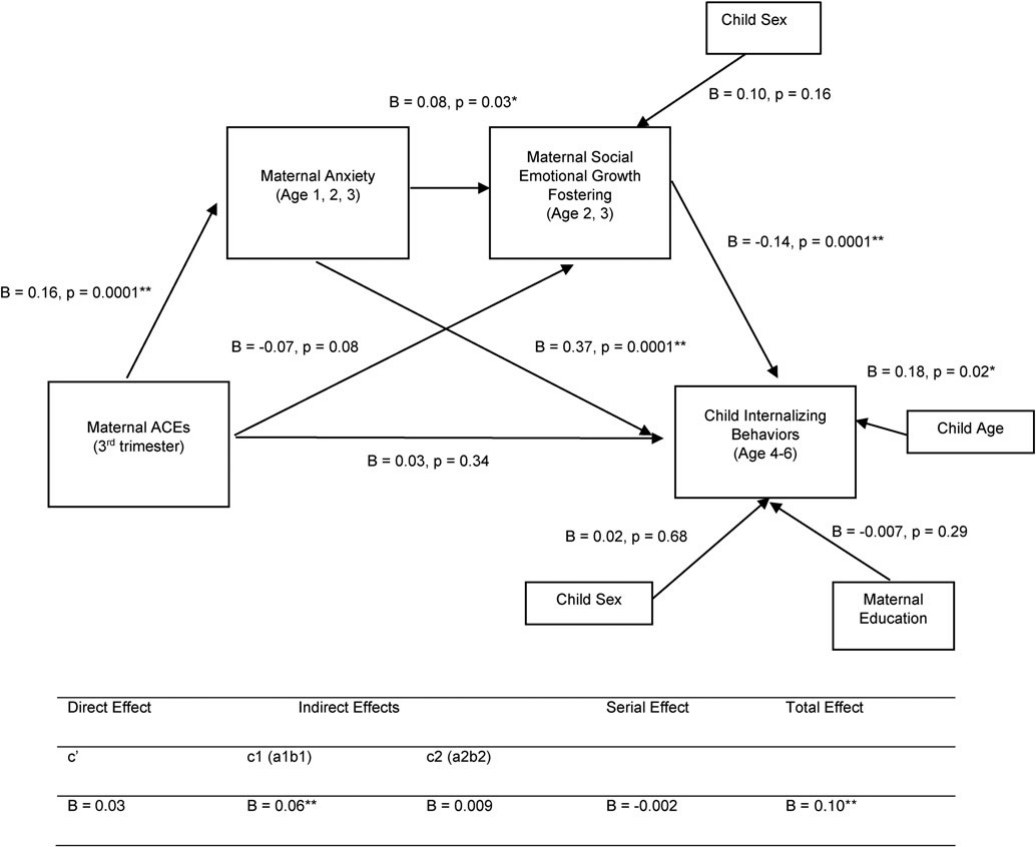
Mediation model. This model was conducted on the analytical sample (*N* = 1030), with variables imputed via FIML. Standardized path coefficients are reported. Controlling for child age, sex, and maternal education, results indicate a significant indirect effect from maternal ACEs to child internalizing problems through maternal anxiety. *p < 0.05; **p < 0.01; ***p < 0.001. ACEs, adverse childhood experiences.

To examine specific positive PCIs akin to parental scaffolding behaviors, we estimated two additional models which included cognitive growth fostering (Fig. 3) and social-emotional growth fostering (Fig. 4) as potential mediators of the maternal ACEs and child internalizing problems association. For the model with cognitive growth fostering included as a mediator, model fit was adequate; SRMR = 0.03, RMSEA = 0.05 [0.03–0.08], CFI = 0.92 (Fig. 3). The indirect effect of maternal ACEs on children's internalizing via a pathway of maternal anxiety was significant (*B* = 0.06, s.e. = 0.01, *p* < 0.001 [0.03–0.09]), as was the indirect effect via cognitive growth fostering (*B* = 0.01, s.e. = 0.005, *p* = 0.049 [0.0001–0.02]). However, there was no evidence that the association between maternal ACEs and children's internalizing behaviors was mediated by a pathway of maternal anxiety to cognitive growth fostering (*B* = −0.001, s.e. = 0.001, *p* = 0.109 [−0.003, 0.0001]) (i.e. no serial mediation). The full model accounted for 15% of the variance in internalizing behaviors.

For the model with social-emotional growth fostering included as a mediator, model fit was also adequate; SRMR = 0.03; RMSEA = 0.04 [0.02–0.07]; CFI = 0.95 (Fig. 4). The indirect effect of maternal ACEs on children's internalizing via a pathway of maternal anxiety was also significant (*B* = 0.06, s.e. = 0.01, *p* < 0.001 [0.03–0.09]). However, the indirect effect via social-emotional growth fostering (*B* = 0.01, s.e. = 0.006, *p* = 0.102 [−0.002–0.02]) was not significant. Results of the serial mediation test (*B* = −0.002, s.e. = 0.001, *p* = 0.075 [−0.004 to 0.0001]) were also not significant. The full model accounted for 16% of the variance in internalizing behaviors.

## Discussion

We examined the intergenerational associations between maternal ACEs and childhood internalizing behaviors and the extent to which maternal anxiety and positive PCIs mediated these associations in a large, diverse, pregnancy cohort study. We found that maternal ACEs were positively associated with child internalizing behaviors at age 4-to-6, with a small but significant effect (hypothesis 1). In addition, both maternal anxiety and positive PCIs related to cognitive growth fostering assessed during toddlerhood independently mediated this association (hypothesis 2). Standardized effect sizes for the indirect effects suggest that the relative contribution of maternal anxiety (*B* = 0.06) on later childhood internalizing behaviors was greater than the relative contribution of positive PCIs related to cognitive growth fostering (*B* = 0.01), with approximately 15% of overall variance accounted for by the model. However, the tests of serial mediation were not significant, suggesting that the influence of maternal ACEs on children's internalizing may not operate through a more complex sequential pathway of maternal anxiety to positive PCIs (hypothesis 3). Overall, findings expand our understanding of the role of maternal mental health in the intergenerational associations between maternal ACEs and their children's emotional and behavioral problems (Folger et al., 2018; Letourneau et al., 2019; McDonald et al., 2019; Stepleton et al., 2018) to include pathways through maternal anxiety and observed PCIs specifically. Our findings also add to the literature, providing new evidence for maternal cognitive growth fostering behaviors as a mediator between maternal ACEs and children's adjustment.

Consistent with most prior literature, we found a small association between maternal ACEs and subsequent anxiety in adulthood (Bridgett, Burt, Edwards, & Deater-Deckard, 2015; Moe, von Soest, Fredriksen, Olafsen, & Smith, 2018; Stepleton et al., 2018), and a moderate association between maternal anxiety and child internalizing behaviors (Rees, Channon, & Waters, 2019; Turner, Beidel, Roberson-Nay, & Tervo, 2003; Woodruff-Borden et al., 2002). In contrast to the current findings, one recent longitudinal study did not find evidence to support maternal postnatal anxiety as a mediator for maternal ACEs and child internalizing behaviors (Letourneau et al., 2019). This could be due, in part, to differences in sample demographics, the timing of child outcome measurement, and underlying rates of maternal ACEs and other adversities (i.e. the Alberta study used a primarily White, higher socioeconomic status (SES) sample), underscoring the importance of studying these relations in diverse populations. Our study suggests that broader implementation of programs that support mothers with anxiety (e.g. cognitive behavioral therapy, acceptance and commitment therapy) (Arch & Craske, 2008; Zamani, Zamani, & Habibi, 2019) in populations similar to our study sample may not only benefit the mothers themselves, but also have additional benefits for her children (Hagan et al., 2017).

Our study is one of few to demonstrate that the impact of maternal ACEs on child behavior is mediated by specific positive PCIs. Most prior studies on parenting in the context of ACEs have focused on attachment (Cooke et al., 2019); thus, our findings expand this research to encompass other important, modifiable aspects of PCIs. Associations were strongest for maternal cognitive growth fostering behaviors – which is related to activities such as attention-reallocation and facilitating language development (Graff et al., 2017). Mothers with high levels of early adversity might be more challenged to provide appropriate monitoring, structure, and support for their children. Our simple mediation results with cognitive growth fostering, but not social-emotional growth fostering, suggest that a mother's ability to scaffold her child's cognitive development and self-regulatory capacity may be a key pathway linking maternal ACEs and child internalizing behaviors. Maternal ACEs influence a mother's parenting self-efficacy (Treat, Sheffield-Morris, Williamson, & Hays-Grudo, 2019), which in turn might affect her ability to patiently scaffold her child's learning during a stressful dyadic task. In addition, self-regulation is likely a transactional process between mothers and their children; mothers with a history of ACEs might struggle with regulating their own emotions, which could in turn affect their child's ability to self-regulate, leading to increased emotional and behavioral problems (Choe, Olson, & Sameroff, 2013; Roubinov, Epel, Adler, Laraia, & Bush, 2019). This transactional process may be particularly salient in early childhood, when children are strongly influenced by the family environment and mothers more actively help their children navigate negative experiences through co-regulatory behaviors (Feldman, 2003). Taken together, our study highlights a specific, understudied aspect of parenting, cognitive growth fostering, which may be improved through parenting interventions and ultimately improve child outcomes.

In contrast to our hypotheses, we found a positive, direct association between maternal anxiety and parenting, such that increasing maternal anxiety was associated with more positive PCIs across all three models. Although many studies have found support for the association between maternal anxiety and poorer parenting behaviors, research is mixed with other studies showing weak associations (Seymour, Giallo, Cooklin, & Dunning, 2015) or no associations (Drake & Ginsburg, 2011; Ginsburg et al., 2005; Muzik et al., 2017). One recent study found that mothers with symptoms of post-traumatic stress disorder (PTSD) displayed more responsive parenting that mitigated the negative effects of maternal PTSD symptoms on child functioning (Greene, McCarthy, Estabrook, Wakschlag, & Briggs-Gowan, 2020). Our findings also suggest that some amount of maternal anxiety might be associated with more positive PCIs. It is also possible that the discrepancies in the literature may be due to differences between clinical and non-clinical samples of mothers. For example, clinically anxious mothers are more likely to engage in higher levels of criticism and lower levels of granting of autonomy compared to non-clinically anxious mothers (Ginsburg et al., 2005). Additional research examining positive PCIs in clinical samples is warranted. Our models testing for serial mediation by maternal anxiety and any of our three measures of parenting were not significant (hypothesis 3).

### Limitations

Our study has some limitations that warrant attention. The social-emotional growth fostering subscale had poor scale reliability, which may have limited our ability to detect mediation; thus, these results should be interpreted with caution. Another limitation is that our three-item measure of ACEs does not capture the full range of adverse experiences, such as neglect or divorce. Although its focus on exposure to interpersonal traumas is a potential strength given their potency, this narrow measure may have reduced our ability to detect some associations. In addition, we did not assess either father or partner parenting practices, which certainly contributes to a child's emotional development. Regarding informants, mothers reported on both their own mental health as well as that of their child, which introduces the possibility of reporting bias. However, maternal report is a commonly used approach in studies examining early childhood behaviors when child self-report is not an option (Letourneau et al., 2019). Although our study focused on a diverse population in the southern USA that is largely understudied, it is important to acknowledge that findings from the current study might not generalize to populations with different demographic characteristics or from other regions of the country. However, there are many similar populations in the southern USA that are also characterized by lower SES and majority Black racial compositions, to which these findings may generalize. Additionally, because only one examiner was present to live-code parenting behaviors during the parent–child task, inter-coder reliability for participant data cannot be established. However, as mentioned above, inter-coder reliability was established using non-participant data and examiners maintained a reliability of 85% or higher. Finally, we focused on internalizing behaviors as the primary outcome in our analyses due to their public health relevance and a robust prior literature linking these behaviors to the key predictors in our study. Furthermore, we were interested in expanding research on maternal ACEs to multiple parenting mediators, which was better suited to a single outcome approach. However, future research would benefit from examinations of these associations with other child behavior problems (e.g. externalizing behaviors).

## Conclusion

In summary, findings underscore the importance of examining potential pathways through which maternal ACEs might confer risk for offspring mental health. Our findings highlight the roles that maternal anxiety and malleable aspects of parenting may play in mediating the intergenerational transmission of maternal ACEs to child internalizing behaviors. There are multiple opportunities for screening, assessment, and earlier intervention for healthcare providers to consider that might help break the intergenerational cycle of risk associated with ACEs (Koita et al., 2018). Intervening after a child (or adult) has experienced adversity or trauma may not only help their future well-being, but also be protective for their children. Although screening for ACEs within pediatric populations is a critical emerging effort in healthcare (California Proposition 56 of 2016, 2017), screening parents as well may also be indicated. Findings additionally support the importance of on-going efforts to increase training of medical and mental health professionals to not only screen for ACEs, but also provide trauma-informed care (Oral et al., 2016). Future studies would benefit from assessing the impact of parental ACEs screening and treatment within the context of their children's emotional problems (Biel, Tang, & Zuckerman, 2020). Further research into different aspects of parenting practices could also help facilitate targeted interventions for parents with a history of adversity. Collectively, evidence suggests that prenatal screening for maternal ACEs is indicated for expectant mothers to ensure that mothers receive support around their own mental health and parenting practices; current research, including the present findings, suggest that these policies may benefit mothers exposed to ACEs and reduce behavior problems in the next generation of children.

## Supplementary material

For supplementary material accompanying this paper visit https://doi.org/10.1017/S0033291721001203. click here to view supplementary material
