# Supplementary material for: A path model examination: maternal anxiety and parenting mediate the association between maternal adverse childhood experiences and children's internalizing behaviors
Source: Psychol Med. 2021 May 18;53(1):112–22. doi: 10.1017/S0033291721001203 (PMC9290334; doi:10.1017/S0033291721001203)
Supplement: Supplementary file 1 [file S0033291721001203sup001.docx]

|  | Enrollment | 3^rd^ trimester | Birth | Age 1 | Age 2 | Age 3 | Age 4-6 |
| --- | --- | --- | --- | --- | --- | --- | --- |
| Maternal ACES (Traumatic Life Events Questionnaire) |  | 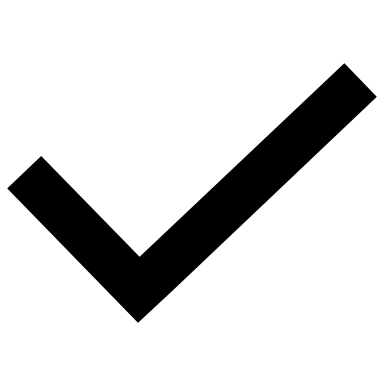 |  |  |  |  |  |
| Maternal Anxiety (Brief Symptom Inventory) |  |  |  | 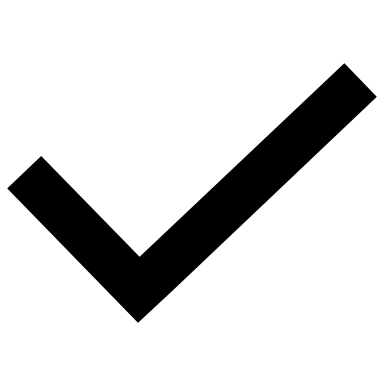 | 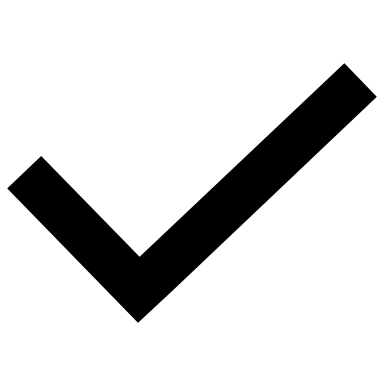 | 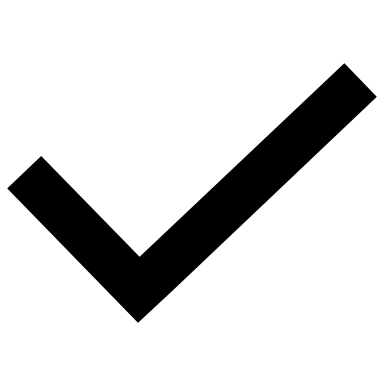 |  |
| Parenting (NCAST Parent-Child Interaction Teaching Scale) |  |  |  |  | 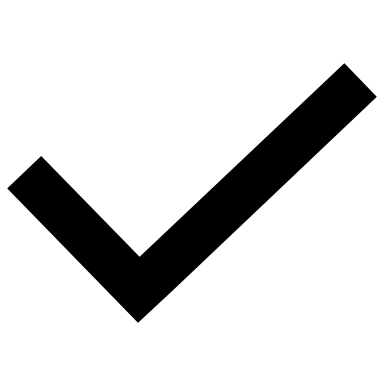 | 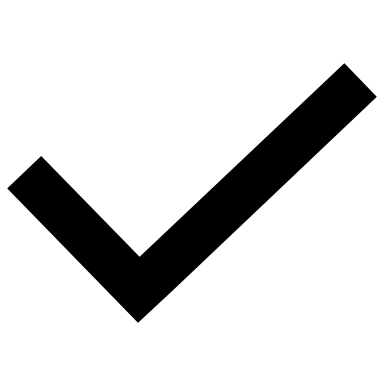 |  |
| Child Internalizing (Child Behavioral Checklist) |  |  |  |  |  |  | 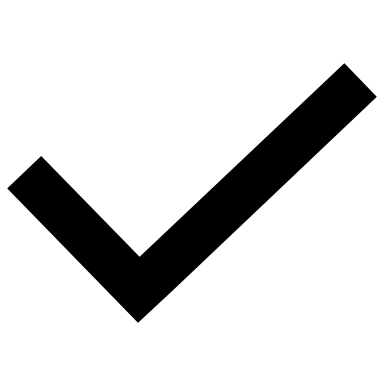 |

**Supplemental Table 1.** CANDLE Study timeline with assessment points and relevant primary variables
